# Probiotic potential of lactic acid bacteria obtained from fermented curly kale juice

**DOI:** 10.1007/s00203-020-02095-4

**Published:** 2020-10-26

**Authors:** Julia Szutowska, Daniela Gwiazdowska

**Affiliations:** Department of Natural Science and Quality Assurance, Institute of Quality Science, Poznań University of Economics and Business, Poznań, Poland

**Keywords:** Non-dairy fermented juice, Spontaneous fermentation, MALDI-TOF spectrometry, Antimicrobial properties, Antibiotic susceptibility assay, Functional properties

## Abstract

The aim of the paper was to analyse changes in lactic acid bacteria (LAB) populations during spontaneous fermentation of green curly kale juice (*Brasicca oleracea* L. var. *acephala* L.) and to determine the probiotic potential of LAB isolates. The analyses revealed that changes in LAB populations were specific for spontaneously fermented vegetable juices. The initial microbiota, composed mostly of *Leuconostoc mesenteroides* bacteria, was gradually replaced by *Lactobacillus* species, mainly *Lactobacillus plantarum, Lactobacillus sakei*, and *Lactobacillus coryniformis.* Screening tests for the antimicrobial properties and antibiotic susceptibility of isolates allowed for the selection of 12 strains with desirable characteristics. *L. plantarum* isolates were characterized by the widest spectrum of antimicrobial interactions, both towards Gram-positive and Gram-negative bacteria. Also, *L. plantarum* strains exhibited the best growth abilities under low pH conditions, and at different NaCl and bile salt concentrations. All strains showed different levels of antibiotic sensitivity, although they were resistant to vancomycin and kanamycin. The present study has shown that bacterial isolates obtained from spontaneously fermented kale juice could constitute valuable probiotic starter cultures, which may be used in fermentation industry.

## Introduction

In recent years, the number of studies involving the isolation of autochthonous plant microorganisms, especially LAB, and determination of their functional properties has been growing, both in the fermented food industry and in scientific research. In this context, many scientific explorations involved intensive investigation of novel fermented plant materials, such as curly kale, cactus pear, red dragon fruit, or cherry, as well as traditional fermented foods, e.g. kimchi or sauerkraut. Both novel and traditional plant materials constitute promising sources of new unique probiotic candidates (Beganović et al. 2014; Filannino et al. 2015; Michalak et al. 2018; Verón et al. 2019; Yien Ong et al. 2012). Basically, autochthonous microorganisms may be isolated from raw, unfermented plants, as well as during different stages of spontaneous fermentation. The population of microorganisms is characteristic for a specific plant niche and depends on numerous factors like weather, geographical location, harvesting conditions, or plant maturity stage (Lund 1992). The process of spontaneous fermentation also leads to significant changes in the microbial population, during which anaerobic microorganisms, mainly LAB and yeasts, displace aerobic ones (Di Cagno et al. 2013; Hutkins 2019). Importantly, fermented plants allow for the isolation and selection of more diverse microorganisms, which creates greater possibilities in the search of strains with the desired properties, compared to raw materials (Torres et al. 2020). Usually, the genera *Leuconostoc* and *Weisella* dominate the raw plant materials in the first hours of the spontaneous lactic acid fermentation process. At later stages, they are successively replaced by bacteria of the genus *Lactobacillus* (Di Cagno et al. 2013). Accordingly, the strains most frequently isolated from fresh and fermented fruits and vegetables belong to both hetero-fermentative and homo-fermentative species of *Leuconostoc*, *Weisella*, *Pediococcus*, *Lactobacillus* or *Enterococcus*, with a particularly high prevalence of *Lactobacillus plantarum* (Szutowska 2020; Torres et al. 2020; Viridiana et al. 2018).

Spontaneous fermentation and back-slopping, which extend the shelf life of fruits and vegetables in an efficient and cost-effective way, remain the most popular home food preservation methods worldwide (Hutkins 2019). However, industrial-scale production of fermented foods requires the use of identified and widely-tested starter cultures. The use of a carefully selected autochthonous starter culture in the fermentation process provides a number of advantages, like microbiological safety, process repeatability, as well as high nutritional and sensory quality (Di Cagno et al. 2013; Hutkins 2019; Vera-Pingitore et al. 2016). Furthermore, indigenous microorganisms, due to their individual adaptability to the particular plant niche, might be more effective in the process and contribute to a significant increase of important biologically active compounds, compared to commercial starter cultures available on the market (Di Cagno et al. 2013; Filannino et al. 2018; Gobbetti et al. 2010; Torres et al. 2020; Vera-Pingitore et al. 2016). Valuable starter cultures should meet several criteria concerning both the technological process and their probiotic potential (de Melo Pereira et al. 2018; FAO and WHO 2006). Generally, from the technological and economic perspective, they should have the ability to grow rapidly in plant matrices at room temperature (without heating or cooling), to acidify the environment quickly, and to produce desirable aroma compounds and exopolysaccharides that improve consumer perception and acceptance. Due to product safety requirements, the starter cultures should effectively inhibit the growth of spoilage microorganisms or food-borne pathogens, degrade antinutritional components, and not affect the increase in biogenic amines. In addition, ideal microbial isolates should be able to change the concentration or bioavailability of the desired biologically active compounds, like vitamins, phenolic compounds, amino acids, and minerals. At the same time, probiotic properties should comprise antibiotic sensitivity, the ability to survive in the human gastrointestinal tract in adequate amounts (approx. 10^6^–10^7^ CFU/mL), epithelial adhesion capacity, and antimicrobial activity towards pathogens (FAO and WHO 2006). The metabolism of probiotic strains and their by-products, like short-chain fatty acids or gamma-aminobutyric acids, may significantly contribute to the overall health-promoting properties of the final fermented product (Diez-Gutiérrez et al. 2020). As demonstrated so far, the consumption of fermented products may entail such benefits as reducing obesity (Verón et al. 2019), ameliorating type 2 diabetes (Li et al. 2014; Nayak et al. 2011), modulating immune activity (Filannino et al. 2013), and stimulating antitumor activity (Ye et al. 2019).

In response to the above considerations, the aim of this paper was to isolate LAB at different stages of curly kale juice fermentation, and to evaluate the obtained bacterial strains for their probiotic properties. In the first stage of the study, the obtained isolates were screened for antimicrobial properties and antibiotic susceptibility, and identified by MALDI-TOF mass spectrometry. Strains characterized by superior features and species diversity were subjected to an NaCl, acid, and bile salt tolerance assay. Subsequently, the selected isolates were molecularly identified by amplification of the 16S rRNA gene.

## Materials, microorganisms, and methods

### Materials

Fresh, packed shredded green curly kale (*Brasicca oleracea* L. var. *acephala* L.) was purchased from three different local stores in Poznań, Poland. Curly kale leaves were washed and dried. Curly kale juice was obtained using a juice squeezer (Hurom HP, South Korea).

### Indicator microorganisms

The antimicrobial activity of the isolated LAB strains was assessed using strains obtained from the American Type Culture Collection (ATCC) and Polish Collection of Microorganisms (PCM). Gram-positive bacteria included *Bacillus subtilis* PCM 2027, *Listeria monocytogenes* ATCC 1911, *Staphylococcus aureus* ATCC 33862, *Enterococcus faecalis* ATCC 35667 *Micrococcus luteus* ATCC 4698, and *Clostridium perfringens* ATCC 13124; Gram-negative bacteria—*Escherichia coli* ATCC 35218, *Campylobacter jejuni* ATCC 33291, *Salmonella enterica* ser. Enteritidis ATCC 13076, *Yersinia enterocolitica* ATCC 9610, *Pseudomonas aeruginosa* ATCC 9027, *Proteus vulgaris* PCM 542; and yeasts—*Candida albicans* ATCC 10231. The strains were stored in Microbank^Ⓡ^ cryogenic vials (ProLab, Canada) at − 22 °C. Before the experiment microorganisms were propagated on the appropriate medium and incubated at 30 °C (*M. luteus*) and at 37 °C for other microorganisms, for 24 h. For *C. perfringens, E. coli, M. luteus—*Trypticasein Soy Agar (Biocorp, Poland) was used; for *C. jejuni, S. enterica* ser Enteritidis, *Y. enterocolitica, P. vulgaris, L. monocytogenes—*Brain Heart Infusion Agar (Biocorp, Poland); *P. aeruginosa, S. aureus, B. subtilis*—Nutrient Agar (Biocorp, Poland) and for *C. albicans*—Sabouraud Dextrose Agar (Oxoid, Canada).

### Fermentation process

Fermented curly kale juice was prepared by spontaneous lactic acid fermentation with a 1.5% NaCl addition. 1 L of fresh green curly kale juice was divided into sterile Falcon tubes (50 ml) and subjected to the fermentation process. The juice was fermented in the laboratory for 8 days at room temperature (21–23 °C) and then stored for 2 weeks at 4 °C. The samples were collected aseptically after 2, 4, 6, and 8 days of fermentation, and after 2 weeks of storage.

### Isolation of lactic acid bacteria from curly kale juice

Isolation of LAB from fresh and fermented green curly kale juice was conducted using the standard plate method**.** Serially diluted juice samples (0.1 mL) were spread on MRS (Biocorp, Poland) agar plates and incubated at 30 °C for 48 h under anaerobic conditions. Only colonies which displayed different morphologies were chosen. Randomly selected colonies were plated on MRS agar by streaking three times and cultivated for 48 h at 30 °C. Then, single colonies were subcultured in 9 mL of MRS broth for 24 h at 30 °C. After incubation, pellets from the MRS broth cultures were resuspended in fresh broth containing 20% glycerol and stored at − 22 °C for further analyses. All selected isolates were initially examined for Gram staining and catalase activity.

### MALDI-TOF mass spectrometry

The isolated strains were determined using MALDI-TOF Microflex mass spectrometry (Bruker, Germany). LAB isolates were grown on MRS agar plates for 48 h at 30 °C. Cell extraction was performed according to a standard extraction protocol with formic acid (Sigma Aldrich, Germany), provided by Banach et al. (2016). Individual bacterial colonies were transferred to 300 µL of ultra-pure water and mixed, and then 900 µL of ethanol were added. The samples were centrifuged at 13,000 rpm for 2 min. The supernatant was removed, the samples were centrifuged again, and the residual ethanol was removed. Then, the pellets were allowed to dry for 5 min. 25 µL of 70% aqueous formic acid (Sigma Aldrich, Germany), solution was added, and the samples dissolved. 25 µL of acetonitrile (Sigma Aldrich, Germany) were added, and the samples were mixed and centrifuged for 2 min at 13,000 rpm. The supernatant in the amount of 1 µL was spotted on ground steel target plate, and 1 µL of HCCA (a-cyano-4-hydroxycinnamic acid) (Sigma Aldrich, Germany) was applied to each sample. Before the primary measurements, calibration was performed using bacterial test standard *E. coli* DH5α extract. The bacterial spectra were compared to the BioTyper reference library of MALDI-TOF mass spectra and NCBI (The National Center for Biotechnology Information). Values of the identification index ≥ 2.00 were considered high-confidence identification, from 1.70 to 1.99—low-confidence identification, and from 0.00 to 1.69—lack of identification at the species level.

### Antimicrobial properties against pathogens

All isolated strains were tested for antimicrobial activity against Gram-positive bacteria, Gram-negative bacteria, and yeasts (see “Indicator microorganisms”). Microbial suspensions in a sterile saline with an optical density of 0.5 based on McFarland turbidity standard were prepared from 24-h cultures of indicator microorganisms grown on agar slants (according to the description in “Indicator microorganisms”). 1 mL of microbial suspensions were placed on Petri dishes and poured with 20 mL of suitable medium. For *C. perfringens* and *M. luteus* Trypticasein Soy Agar (Biocorp, Poland) was used; for *E. coli, C. jejuni, S. enterica* ser Enteritidis, *Y. enterocolitica, P. aeruginosa, P. vulgaris, L. monocytogenes, S. aureus* and *B. subtilis—*Mueller Hinton Agar (Biocorp, Poland), and for *C. albicans*—Sabouraud Dextrose Agar (Oxoid, Canada). After the medium was solidified, wells (10 mm diameter) were cut out with a sterile cork bore. Then, 24 h bacterial MRS cultures (30 °C) were screened for inhibitory activity using the well diffusion method. An initial inoculum of approximately 1 × 10^8^ CFU/mL (100 µl) of LAB strains was introduced into wells. *M. luteus* plates were incubated at 30 °C and other indicator microorganisms at 37 °C for 24 h. Anaerobic conditions of incubation were provided for *C. jejuni* and *C. perfringens.* In order to obtain the results, the diameter of the inhibitions zones was measured taking into account the well diameter. The study was carried out in three parallel replications. Results were presented as an average from three replications.

### Antibiotic susceptibility test

Antibiotic susceptibility tests of the all isolated strains were performed using the agar disc-diffusion method according to Rubio et al. (2014) with some modifications. Saline suspensions were prepared from 24-h cultures of bacterial strains grown in MRS broth at 30 °C. Optical density was set at 1.0 on the McFarland scale (which refers to 1.5 × 10^7^ CFU/mL). Then, 1 mL of each isolate was poured with 20 mL of MRS agar. Eleven antibiotic discs (Oxoid, Great Britain) were used for the assay: ampicillin (10 µg), gentamicin (10 µg), erythromycin (15 µg), chloramphenicol (30 µg), streptomycin (300 µg), penicillin G (1.5 IU), clindamycin (2 µg), vancomycin (30 µg), tetracycline (30 µg), kanamycin (30 µg), and neomycin (30 µg). Samples were incubated at 30 °C for 48 h. The study was carried out in three parallel replications. Results were presented as an average from three replications, and classified as resistant—*R* (≤ 14 mm), intermediate susceptibility—*I* (15–19 mm), and susceptibility—*S* (≥ 20 mm) for antibiotic discs. The interpretation of sensitivity patterns of tested isolates against standard antibiotics were performed according to the Clinical and Laboratory Standard Institute (CLSI 2019).

### NaCl, acid, and bile salt tolerance

LAB isolates were cultivated on MRS broth for 24 h under anaerobic conditions (10^8^ CFU/mL) at 30 °C. 2 mL of each culture were harvested by centrifugation—8000 rpm for 5 min (MiniSpin Plus, Eppendorf, Germany) and washed twice in 2 mL of phosphate buffer. Then, the bacterial suspensions were further tested for NaCl, acid, and bile salt tolerance. The impact of pH, NaCl, and bile salts on the survival of LAB strains was assessed using microplates. The appropriate pH values of MRS medium were set at pH 2 and 3 with HCl (Sigma Aldrich, Germany), and control was established at pH 6.5. Tolerance to NaCl was examined in the presence of 2, 4, 6 and 8% of sodium chloride. Independently, the level of bile salts (Ox-Bile, Oxoid) has been set at 1%, 0.5% and 0.25%. In all these assays, microplates were incubated for 24 h at 30 °C. Spectrometric measurements were carried out using a BioTek Epoch device (United Kingdom) at a wavelength of 600 nm.

### 16S rRNA gene sequencing and PCR amplification

Molecular identification of LAB strains was performed by amplification of the 16S rRNA gene. First, total genomic DNA of isolates was extracted using the Genomic Mini AX Bacteria + Spin kit (A&A Biotechnology, Poland), according to the manufacturer’s protocol. Subsequently, amplification of the 16S rRNA bacterial gene was conducted by PCR using two universal, degenerate primers: S-D-BACT-0008 (27F) (5′-AGAGTTTGATCCTGGCTCAG-3′) and 1492r (5′-GGTTACCTTGTTACGACTT-3′) (Leite et al. 2015). The subsequent stages of PCR included: 1. initial denaturation at 94 °C for 120 s; 2. 40 cycles consisting of denaturation at 94 °C for 30 s, binding primers at 45 °C for 30 s, and elongation at 72 °C for 120 s; and 3. the final cycle including denaturation at 95 °C for 30 s, binding primers at 45 °C for 30 s, and elongation at 72 °C for 120 s. The amplicons were separated on 1% (w/v) agarose gel with the addition of MIDORI Green (3 μl) by electrophoresis. Amplified DNA fragments were observed under UV light (Omega Lum G, Aplegen, USA). Nucleotide sequencing was performed by Genomed S. A. (Warsaw, Poland). Sequences of approximately 1500 nt length were edited, combined, and generated in GeneDoc 2.700. Finally, homologous sequence searching was conducted using the BLAST algorithm (https://blast.ncbi.nlm.nih.gov/). The unrooted phylogenetic tree was constructed to determine the closest bacterial species by the neighbour-joining method (Saitou and Imanishi 1989) using the MEGA X software (Kumar et al. 2018). To construct the phylogenetic tree, 16S rRNA reference sequences of *L. mesenteroides* (GenBank ID: MN796044; MT572967), *L. miyukkimchi* (GenBank ID: NR_109072)*, L. gelidum* (GenBank ID: LC306838)*, L. inhae* (GenBank ID: LC519999)*, L. sakei* (GenBank ID: MT626078), *L. plantarum* (GenBank ID: AB572048; MG646838; MT538586) and *L. coryniformis* (GenBank ID: MT211344) were obtained from NCBI database.

## Results and discussion

### Isolation and identification of lactic acid bacteria by MALDI-TOF mass spectrometry

A total of 80 LAB isolates were obtained both from fresh and spontaneously fermented green curly kale juice. In the first step, macro- and microscopic evaluation, Gram staining, and catalase-producing ability were used to confirm that the isolated bacteria belong to the group of LAB. Both rods and cocci were found to belong to Gram-positive bacteria, and all were marked as catalase-negative.

Subsequently, an attempt was made to examine changes in LAB population dynamics during spontaneous green curly kale juice fermentation. All isolates were identified by fingerprinting of cell ribosomal proteins (MALDI-TOF MS). Summary of isolates identification is presented in Table 1. The study revealed that fresh and 2-day fermented curly kale juice were characterized by the presence of *Leuconostoc* species, mainly *Leuconostoc mesenteroides.* Metabolism of *Leuconostoc* and *Weisella* species during spontaneous fermentation of cruciferous vegetables is pivotal for the success of the whole process. At the initial stage of fermentation (often called the heterolactic or gaseous phase), these bacteria are capable of significantly lowering the pH of the product, producing CO_2_ in appropriate amounts, and therefore inhibiting the growth of non-lactic or spoilage microbiota (Hutkins 2019). As shown in our previous studies on fermented curly kale juice (Szutowska et al. 2020), after 48 h of spontaneous fermentation, obligate aerobic microorganisms and fungi decreased, while the number of LAB, enterococci, and yeasts increased significantly. Thus, the initial microbiota activity resulted in the creation of an optimal environment for other microorganisms, responsible for stronger acidification of the product and the successful completion of the fermentation process (Szutowska et al. 2020). In the subsequent stages of fermentation and storage, a decrease in the *Leuconostoc* population corresponded with the succession of *Lactobacillus* species, notably *L. plantarum, L. coryniformis*, and *L. sakei*. As was shown in our previous research, a drop in pH value from 5.08 on day 2 to 4.08 on the last day of fermentation was observed (Szutowska et al. 2020). Therefore, it can be concluded that, bacteria of the genus *Lactobacillus* acted as strong acid producers.

**Table 1:** MALDI-TOF identification summary of isolates

Stage of fermentation	No. of isolates	Morphology	MALDI-TOF identification
Fresh juice	13	Cocci	*L. mesenteroides*
	2	Cocci	No identification possible
2nd day of fermentation	13	Cocci	*L. mesenteroides*
4th day of fermentation	9	Rods	*L. sakei, L. plantarum* and *L. coryniformis*
	2	Cocci	No identification possible
	2	Cocci	*L. mesenteroides*
6th day of fermentation	13	Rods	*L. plantarum* and *L. coryniformis*
8th day of fermentation	13	Rods	*L. plantarum* and *L. coryniformis*
Storage (2 weeks)	13	Rods	*L. plantarum, L. coryniformis, L. coryniformis* subsp. *torquens*

Michalak et al. (2018) observed similar changes in the population dynamics of LAB throughout the process of spontaneous curly kale fermentation. In addition to *Leuconostoc* and *Lactobacillus* species, the researchers identified bacteria of the genera *Weisella*, *Pediococcus*, and *Lactococcus* during various stages of fermentation. However, compared to the present study, the cited authors (Michalak et al. 2018) isolated different strains of the genus *Lactobacillus* (with the exception of *L. plantarum*), like *L. curvatus, L. paraplantarum,* and *L. brevis*. Moreover, the obtained results were consistent with findings by other authors (Xiong et al. 2012), who studied the spontaneous fermentation of Chinese sauerkraut, and reported that the process was initiated by *L. mesenteroides* subsp. *mesenteroides*, which were successively replaced by *E. faecalis, L. lactis, L. zeae, L. plantarum*, and *L. casei* as fermentation progressed*.*

In general, the species *L. mesenteroides, L. plantarum, L. sakei*, and *L. coryniformis* isolated in this study belong to the typical microbiota of spontaneously fermented vegetables. Based on multiple research reports, individual strains of the above-mentioned species can be used as potential starter cultures in fermented food production technology. For example, members of the genus *Leuconostoc*, primarily *L. mesenteroides*, are widely used in the fermented food industry as a single culture or as part of microbial community starters, especially in the commercial production of kimchi (Park et al. 2019). Their popularity and frequency of usage is strictly associated with specific features like exopolysaccharide content or the production of desirable flavour and aroma compounds (Hutkins 2019; Li et al. 2020). In turn, *L. plantarum* is one of the most commonly identified and isolated LAB species in different kinds of fermented foods (like kimchi, sauerkraut, pickles, or olives) (Beganović et al. 2014; Di Cagno et al. 2009; Hutkins 2019; Xiong et al. 2012). Due to its remarkable adaptability to various environment conditions, strains of *L. plantarum* might be characterized by numerous probiotic properties, such as cholesterol lowering activity, diarrheal prevention, or management of gastrointestinal disorders (Filannino et al. 2016; Seddik et al. 2017). For this reason, *L. plantarum* strains are frequently used as starter cultures for carrying out controlled fermentation of both traditional and new products (Szutowska 2020; Torres et al. 2020). Literature data indicate that strains of *L. sakei* are able to dominate diversified environments of spontaneously fermented foods, from sauerkraut or kimchi to meat and sake products (Champomier-Vergès et al. 2002; Eisenbach et al. 2019). What is fundamental, the strains of *L. sakei* play a crucial role in the preparation of kimchi, especially in the synthesis of B vitamins, like thiamine or folate, and in the inability to produce biogenic amines or toxins (Kim et al. 2020). Therefore, the isolated *L. sakei* JS034 strain most likely forms an important part of the microbial population during curly kale juice fermentation, and might be used as a valuable starter culture in future investigations. Despite the fact that bacteria of the species *L. coryniformis* are also natural members of many spontaneously fermented products, similarly to *L. plantarum* or *L. sakei,* they are less often isolated and used as a potential starter culture. A few studies (Fang et al. 2016; Magnusson and Schnürer 2001; Martín et al. 2005) demonstrated that *L. coryniformis* possessed promising and appealing features based on the broad spectrum of antimicrobial compounds they produce, like proteinaceous antifungal compounds or bacteriocin-reuterin, and their ability to eliminate dietary carcinogens (e.g. nitrites). However, due to insufficient studies on *L. coryniformis* in fermented foods, the research gap concerning its functional and technological characteristics as a potential starter culture remains.

### Antimicrobial properties of isolates

**Fig. 1:**
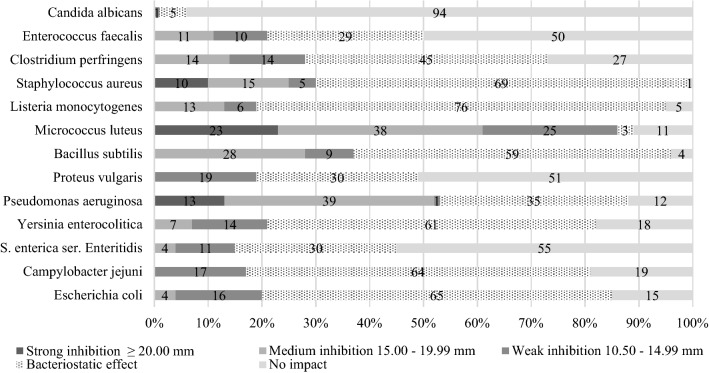
Percentages of isolates showing antimicrobial properties

In order to determine their functional and probiotic properties, all 80 LAB isolates were examined for antimicrobial activity against Gram-positive, Gram-negative, and yeast indicator microorganisms (Fig. 1). Antimicrobial properties, together with the antibiotic susceptibility assay results, enabled the selection of microorganisms with desired characteristics for further investigations. In the analysed strains, strong and moderate antimicrobial properties were mainly observed against Gram-positive bacteria. *S. aureus, B. subtilis,* and *M. luteus* proved to be particularly sensitive. In relation to Gram-negative pathogens, the tested isolates mainly showed weak inhibition properties or even bacteriostatic activity. The only exception was the *P. aeruginosa* strain*,* which proved relatively susceptible to LAB isolates and their metabolites. Generally, the microorganisms most resistant to the influence of the isolates were *P. vulgaris* bacteria and *C. albicans* yeasts*.*

The antimicrobial properties of starter cultures are one of the most essential functional features necessary for determining the probiotic characteristics of the isolated strains. On the basis of screening tests (Fig. 1) concerning the antimicrobial potential of LAB isolates, 12 strains (Table 2) characterized by superior antimicrobial activity were selected. *Lactobacillus* strains (especially: JS034, JS052, JS053, JS058, and JS075) demonstrated significant antimicrobial activity against most indicator microorganisms, especially in relation to Gram-positive bacteria like: *B. subtilis, S. aureus, M. luteus, L. monocytogenes* and Gram-negative bacteria—*P. aeruginosa*. Meanwhile, bacteria of the genus *Leuconostoc* exhibited a slightly lower antimicrobial potential in general, with the exception of indicator strains of *E. faecalis, S.* Enteritidis, and *P. vulgaris,* for which they did not manifest antagonistic activity. Tamang et al. (2009) likewise demonstrated that *Lactobacillus* strains possessed stronger antimicrobial properties compared to *Leuconostoc* species. In addition, they showed that in general, isolates derived from ethnic fermented vegetables and tender bamboo shoots exhibited a satisfactory antagonistic activity against a wide spectrum of pathogens, including *L. monocytogenes, S. aureus, S. mutans*, and *P. aeruginosa.*

**Table 2:** Growth inhibition of indicator microorganisms by selected LAB strains

Indicator microorganism	Zone of inhibition (diameter in mm)											
	JS001	JS017	JS024	JS027	JS031	JS032	JS034	JS052	JS053	JS058	JS070	JS075
*B. subtilis*	13.3 ± 0.6	11.7 ± 0.6	14.7 ± 0.6	16 ± 0.0	15.33 ± 0.6	18 ± 0.0	17 ± 0.0	19 ± 0.0	18 ± 0.0	17.3 ± 0.6	14 ± 0.0	17.6 ± 0.6
*L. monocytogenes*	15 ± 0.0	18.5 ± 0.5	16.3 ± 0.6	20.6 ± 0.6	17 ± 0.0	18.3 ± 0.6	15.5 ± 0.5	15.5 ± 0.5	18.6 ± 0.6	15 ± 0.0	0	19 ± 0.0
*S. aureus*	15.3 ± 0.6	14 ± 0.0	13 ± 0.0	14.6 ± 0.6	12.5 ± 0.5	15.5 ± 0.5	19.6 ± 0.6	19.6 ± 0.6	19.5 ± 0.5	18 ± 0.0	22.5 ± 0.5	15.5 ± 0.5
*E. faecalis*	0	0	0	0	13.5 ± 0.5	0	17.3 ± 0.6	16.3 ± 0.6	13.6 ± 0.6	11.3 ± 0.6	0	0
*M. luteus*	14.5 ± 0.5	13.5 ± 0.5	11 ± 0.0	13 ± 0.0	14.6 ± 0.6	15.6 ± 0.6	23.3 ± 0.6	21 ± 0.0	21 ± 0.0	21.5 ± 0.5	20.3 ± 0.6	21.3 ± 0.6
*C. perfringens*	11.6 ± 0.6	11 ± 0.0	0	13.3 ± 0.6	13.5 ± 0.5	14 ± 0.0	15.5 ± 0.5	13.6 ± 0.6	14.6 ± 0.6	13.3 ± 0.6	0	13.5 ± 0.5
*E. coli*	12 ± 0.0	12 ± 0.0	0	12.3 ± 0.6	13.6 ± 0.6	13.3 ± 0.6	15.6 ± 0.6	14 ± 0.0	13.6 ± 0.6	18.5 ± 0.5	11 ± 0.0	14.5 ± 0.5
*C. jejuni*	14.3 ± 0.6	18.3 ± 0.6	15 ± 0.0	16.5 ± 0.5	15.3 ± 0.6	15.6 ± 0.6	11.5 ± 0.5	14.5 ± 0.5	13.5 ± 0.5	13 ± 0.0	0	15 ± 0.0
*S. enteritidis*	13 ± 0.0	0	0	0	0	0	14 ± 0.0	15.6 ± 0.6	13.6 ± 0.6	14.5 ± 0.5	0	15.6 ± 0.6
*Y. enterocolitica*	0	0	15 ± 0.0	16.5 ± 0.5	15.5 ± 0.5	15.5 ± 0.5	14.3 ± 0.6	13.3 ± 0.6	14 ± 0.0	15.6 ± 0.6	0	15.3 ± 0.6
*P. aeruginosa*	0	11.6 ± 0.6	14.6 ± 0.6	17 ± 0.0	15.5 ± 0.5	15.6 ± 0.6	17.5 ± 0.5	16.6 ± 0.6	17.6 ± 0.6	16 ± 0.0	21 ± 0.0	17.3 ± 0.6
*P. vulgaris*	0	0	0	0	13 ± 0.0	0	13.3 ± 0.6	12.6 ± 0.6	13.5 ± 0.5	11.5 ± 0.5	15.3 ± 0.6	16.5 ± 0.5
*C. albicans*	0	0	0	0	0	0	0	0	0	0	11.3 ± 0.6	11 ± 0.0

Diameter represented as mean ± SD of triplicate analyses

Apparently, inhibition of microorganism growth by LAB is associated with various metabolites, such as organic acids, diacetyl, bacteriocins, biosurfactants, or carbon dioxide (Reis et al. 2012; Šušković et al. 2010). Lactic and acetic acid production, and thus pH lowering, mainly contributed to the effective inhibition of food-borne pathogen or spoilage microorganism growth in fermented foods (Reis et al. 2012; Šušković et al. 2010). The production of organic acids by LAB can inhibit Gram-negative bacteria through the penetration of cell membrane and therefore affecting its functioning, acidifying cytoplasm and inhibiting acid-sensitive enzymes (Cervantes-Elizarrarás et al. 2019). Another important aspect is the fact that bacterial isolates from later fermentation stages are often characterized by increased organic acid synthesis (Hutkins 2019). Thus, the present analyses indicated that strains derived from more advanced stages of the spontaneous fermentation process of kale juice exhibited stronger antagonistic activity. Probably their ability to significantly acidify the curly kale juice affected their antimicrobial properties. As demonstrated, strains isolated at days 4 and 8 of fermentation (species *L. plantarum* and *L. coryniformis*) contributed to the inhibition of the growth of all indicator organisms (except for *C. albicans*) to a lesser or greater extent*.*

In a study (Patel et al. 2014) on LAB strains isolated from fresh vegetables and traditionally fermented Indian food, the authors noticed distinct antimicrobial properties against *E. coli* and *S. aureus* depending on the strain used. Similar conclusion arisen in the context of this antimicrobial study, which demonstrated that the antagonistic interaction of LAB isolates is also strain-dependent. Moreover, the cited researchers (Patel et al. 2014) observed, as in the present paper, that strains of *L. plantarum* possessed better inhibitory properties compared to other tested isolates. They hypothesized that this was associated with the strains’ bacteriocin production ability. Based on numerous scientific reports (Biswas et al. 2017; Ponce et al. 2008; Yi et al. 2020), it can be assumed that also LAB isolates derived from fresh and fermented curly kale juice may be characterized by the ability to synthesize bacteriocins with a broad spectrum of antimicrobial activity. It seems likely that the strong antimicrobial properties of *L. plantarum* strains (JS034, JS052, JS053) could be correlated with their production of bacteriocins. According to a literature review, (da Silva Sabo et al. 2014) mainly *L. plantarum* strains showed a considerable potential as bacteriocin bio-producers. The antimicrobial mechanism of specific bacteriocin activity might be based on the inhibition of DNA or RNA synthesis, disturbance of membrane potential, and leakage of anions or cations from the bacterial cell (Gwiazdowska and Trojanowska 2005). However, to confirm this assumption, further investigations are required.

### Antibiotic susceptibility assay

**Fig. 2:**
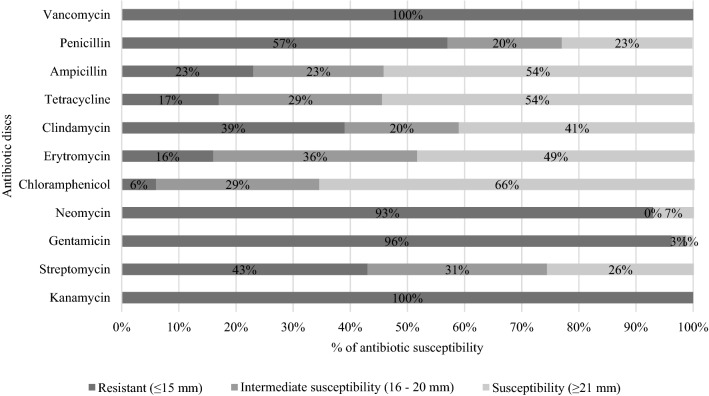
Percentages of isolates showing antibiotic susceptibility

Due to the possibility of antibiotic resistance transfer from food microorganisms like LAB to human pathogens, a basic study was performed to determine the obtained strains’ resistance towards commonly used antibiotics. In this regard, all isolates of LAB have been screened against a broad spectrum of antibiotics that acted as inhibitors of protein synthesis or cell wall synthesis (Fig. 2). Detailed results regarding the susceptibility of the 12 selected potentially probiotic strains to the most common antibiotics are presented in Table 3.

**Table 3:** Antibiotic susceptibility of selected LAB strains

Isolate	Group of antibiotics										
	Inhibitors of protein synthesis								Inhibitors of cell wall synthesis		
	K (30 µg)	S (300 µg)	G (10 µg)	N (30 µg)	C (30 µg)	E (15 µg)	DA (2 µg)	T (30 µg)	AMP (10 µg)	P (1.5 IU)	VA (30 µg)
JS001	R	S	I	I	S	S	S	S	S	I	R
JS017	R	I	R	R	S	S	S	S	S	I	R
JS024	R	R	R	R	S	I	I	I	I	R	R
JS027	R	S	R	I	S	S	S	S	S	S	R
JS031	R	I	R	R	S	S	I	S	S	I	R
JS032	R	I	R	R	I	I	I	S	S	R	R
JS034	R	I	R	R	S	S	R	I	S	R	R
JS052	R	I	R	R	S	S	R	I	I	R	R
JS053	R	I	R	R	I	I	S	S	S	R	R
JS058	R	S	I	R	S	S	S	I	S	I	R
JS070	R	S	R	S	S	S	S	S	S	S	R
JS075	R	S	R	R	S	S	S	S	S	S	R

*K* kanamycin, *S* streptomycin, *G* gentamicin, *N* neomycin, *C* chloramphenicol, *E* erythromycin, *DA* clindamycin, *T* tetracycline, *AMP* ampicillin, *P* penicillin, *VA* vancomycin, *R* resistant, *I* intermediate, *S* sensitive

Based on the antibiotic susceptibility assay, all isolates were resistant to vancomycin, kanamycin and 93% and 96% of LAB strains, respectively, were resistant to neomycin and gentamicin (Fig. 2). These findings are consistent with those from other studies (Argyri et al. 2013; Colombo et al. 2020; Michalak et al. 2018; Nawaz et al. 2011), which also reported that LAB isolated from different environments, like fermented olives, curly kale, or dairy products, were resistant to vancomycin, kanamycin, and others antibiotics. According to literature data (Mathur and Singh 2005; Nawaz et al. 2011) LAB resistance to vancomycin, kanamycin, gentamicin, or neomycin may be an inherent characteristic of the bacterial species or even genus. Therefore, intrinsic resistance cannot be horizontally transferable among microorganisms. Bacteria of the genera *Lactobacillus, Pediococcus*, and *Leuconostoc* have been frequently reported to possess strong natural resistance to the above-mentioned antibiotics (Argyri et al. 2013; Colombo et al. 2020; Michalak et al. 2018; Nawaz et al. 2011). For this reason, such an ability is also seen as a diagnostic and characteristic feature of the LAB group.

Generally, *L. plantarum* strains (JS034 and JS052) were characterized by higher resistance to such antibiotics as kanamycin, gentamicin, neomycin, clindamycin, vancomycin and penicillin compared to other tested isolates. Also *L. mesenteroides* JS024 proved to be particularly resistant towards kanamycin, streptomycin, gentamicin, neomycin, vancomycin and penicillin. Moreover, based on the present analyses and other scientific reports (Mathur and Singh 2005), there are significant differences in antibiotic resistance on the intergenus and interspecies levels. For example, strains of *L. mesenteroides* (JS001 and JS024) showed differentiated susceptibility to streptomycin, and strains of *L. plantarum* (JS052, and JS053) also had diverse levels of susceptibility towards, chloramphenicol, erythromycin, tetracycline, and ampicillin. On the other hand, such antibiotics as chloramphenicol, ampicillin, tetracycline, and erythromycin satisfactorily inhibited the growth of most screened strains. These results are consistent with literature data, which implied that lactobacilli are more susceptible to antibiotics targeting protein synthesis, like tetracycline and streptomycin—which involves weakening the ribosome-tRNA interaction or chloramphenicol and erythromycin responsible for blocking peptidyl-transferase activity (Jose et al. 2015).

### NaCl, acid, and bile salt tolerance

**Fig. 3:**
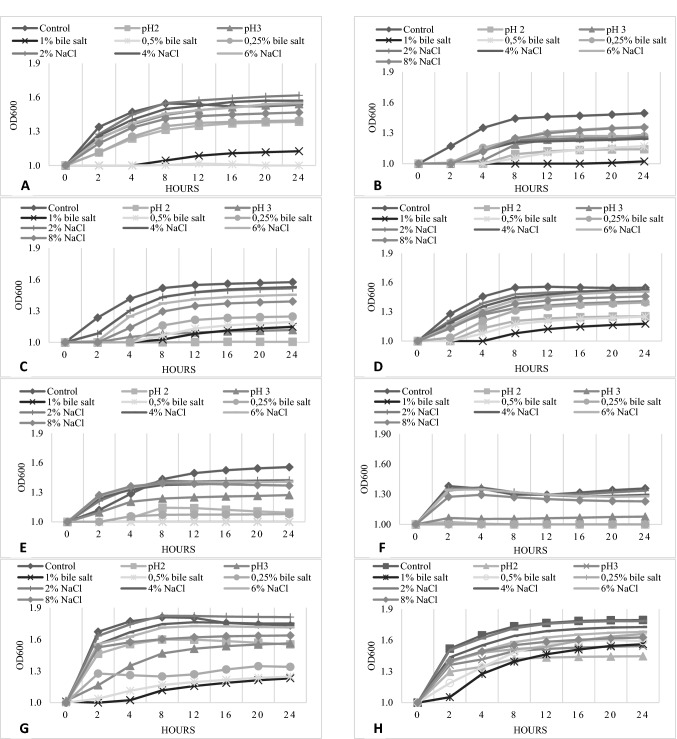

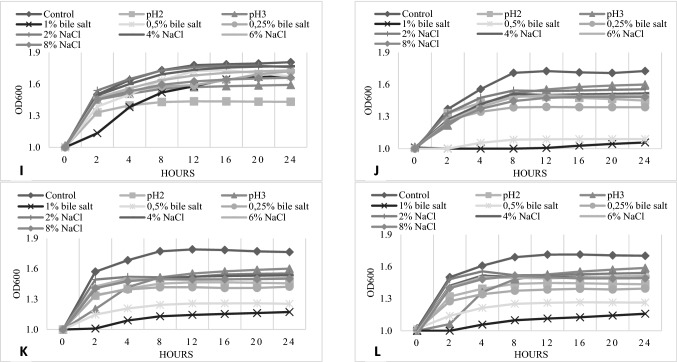
NaCl, acid and bile salt tolerance

The next stage of the present study on the functional properties of selected LAB involved assessing their growth at low pH values and different concentrations of NaCl and bile salts (Fig. 3). Growth at low pH values and at the presence of bile salts is extremely important for the technological usage of bacterial strains on an industrial scale and determination of their probiotic properties—they should survive the conditions of the human digestive tract in sufficient amounts as to be beneficial for the host. Also, good growth and survival of starter culture in the presence of NaCl is a desirable feature due to the frequent use of salt as food preservation in various fermented products (Hutkins 2019). Isolates belonging to the species *L. plantarum* (JS052 and JS053) were characterized by the most appealing growth among other strains under all tested parameters. These superior properties, together with antimicrobial activity and antibiotic susceptibility, constitute desired characteristics in potential candidates for probiotic strains. These findings are consistent with those from other studies concerning isolates from curly kale (Michalak et al. 2018), in which strains of *L. plantarum* and *L. brevis* demonstrated noteworthy tolerance towards low pH values and various NaCl and bile salt concentrations. The authors Lee et al., (2016) also noticed that *L. plantarum* isolate obtained from kimchi, were characterized by better level of resistance against low pH values and 0.3% bile salts compared to other isolates like as *L. mesenteroides.* On the other hand, the strains of *L. mesenteroides, Leuconostoc* spp. (JS017 and JS031)*, L. sakei*, and *L. coryniformis* showed significantly weaker growth, especially in the presence of 6% and 8% NaCl as well as at low pH values. For *L. mesenteroides* JS001, JS017, and JS031, and for *L. sakei*, no growth was observed following the addition of 1% or 0.5% of bile salts.

Interestingly, individual strains of *L. plantarum* (JS034, JS052, and JS053) exhibited satisfactory growth in the presence of 1% bile salts. This might be associated with the ability of the isolates to detoxify bile salts by producing an intracellular bile salt hydrolase enzyme (BSH). However, the presence of the BSH enzyme is mainly identified in the intestinal microbiota (Begley et al. 2006), for which bile salts are a natural component of the habitat. Much less often is it observed in strains from environmental samples, like dairy or fermented foods (e.g. *Boza, Zabady*, or *Rayeb*) (Horackova et al. 2020; Shehata et al. 2016). It is also speculated that *bsh* genes, responsible for BSH enzyme synthesis, could be horizontally transferred among microorganisms (Begley et al. 2006). Additionally, recent scientific studies (Ru et al. 2019) proved that LAB derived from naturally fermented vegetables demonstrated satisfactory bile salt deconjugation properties, despite the absence of bile salt in the vegetable matrix.

### Identification by 16S rRNA gene

Previously selected strains with probiotic potential based on species diversity and functionality were identified with the use of the 16S rRNA gene. Comparative results of mass spectrometry and molecular method are shown in Table 4. According to data in Table 4, the results of LAB identification by two independent methods proved to be comparable and accurate. Notably, both the MALDI-TOF method and genetic identification with the use of 16S rRNA had some limitations. Two strains (JS017 and JS031) derived from fresh and fermented juice have not been identified due to data restrictions in both scientific databases. The sequences of all isolates, excluding the two mentioned here, have been deposited in the GenBank database (Table 4).

**Table 4:** Comparison of identification of 12 selected isolates by MALDI-TOF MS and 16S rRNA gene sequence

Stage of fermentation	Isolate	MALDI-TOF Identification		16S rRNA gene sequencing	
		Results	Log score value	Results	Identification value (%)/reference strain
Fresh juice	JS001	*Leuconostoc mesenteroides*	2.18	*Leuconostoc mesenteroides* (GenBank ID: MT422152)	98.93%/*L. mesenteroides*^*T*^ (GenBank ID: MN796044)
	JS017	No identification possible	1.23	No identification possible	99.58%/*L. miyukkimchi*^*T*^ (GenBank ID: NR_109072)
					99.58%/*L. inhae *^*T*^ (GenBank ID: LC519999)
					99.58%/*L. gelidum*^*T*^ (GenBank ID: LC306838)
Day 2 of fermentation	JS024	*Leuconostoc mesenteroides*	2.42	*Leuconostoc mesenteroides* (GenBank ID: MT434139)	99.09%/*L. mesenteroides*^*T*^ (GenBank ID: MN796044)
	JS027	*Leuconostoc mesenteroides*	2.11	*Leuconostoc mesenteroides* (GenBank ID: MT434342)	99.59%/*L. mesenteroides*^*T*^ (GenBank ID: MT572967)
Day 4 of fermentation	JS031	No identification possible	1.19	No identification possible	98.84%,/*L. miyukkimchi*^*T*^ (GenBank ID: NR_109072)
					98.36%/*L. gelidum*^*T*^ (GenBank ID: LC306838)
	JS032	*Lactobacillus sakei*	2.32	*Lactobacillus sakei* (GenBank ID: MT434340)	99.26%/*L. sakei*^*T*^(GenBank ID: MT626078)
	JS034	*Lactobacillus plantarum*	2.42	*Lactobacillus plantarum* (GenBank ID: MT434007)	98.72%/*L. plantarum*^*T*^(GenBank ID: AB572048)
Day 8 of fermentation	JS052	*Lactobacillus plantarum*	2.30	*Lactobacillus plantarum* (GenBank ID: MT434011)	99.47%/*L. plantarum*^*T*^ (GenBank ID: MG646838)
	JS053	*Lactobacillus plantarum*	2.20	*Lactobacillus plantarum* (GenBank ID: MT425508)	99.50%/*L. plantarum*^*T*^ (GenBank ID: MT538586)
	JS058	*Lactobacillus coryniformis*	2.15	*Lactobacillus coryniformis* (GenBank ID: MT425517)	99.26%/*L. coryniformis*^*T*^ (GenBank ID: MT211344)
Storage	JS070	*Lactobacillus coryniformis* subsp. *torquens*	2.35	*Lactobacillus coryniformis* (GenBank ID: MT378387)	99.60%/*L. coryniformis*^*T*^ (GenBank ID: MT211344)
	JS075	*Lactobacillus coryniformis*	2.07	*Lactobacillus coryniformis* (GenBank ID: MT434141)	99.26%/*L. coryniformis*^*T*^ (GenBank ID: MT211344)

**Fig. 4:**
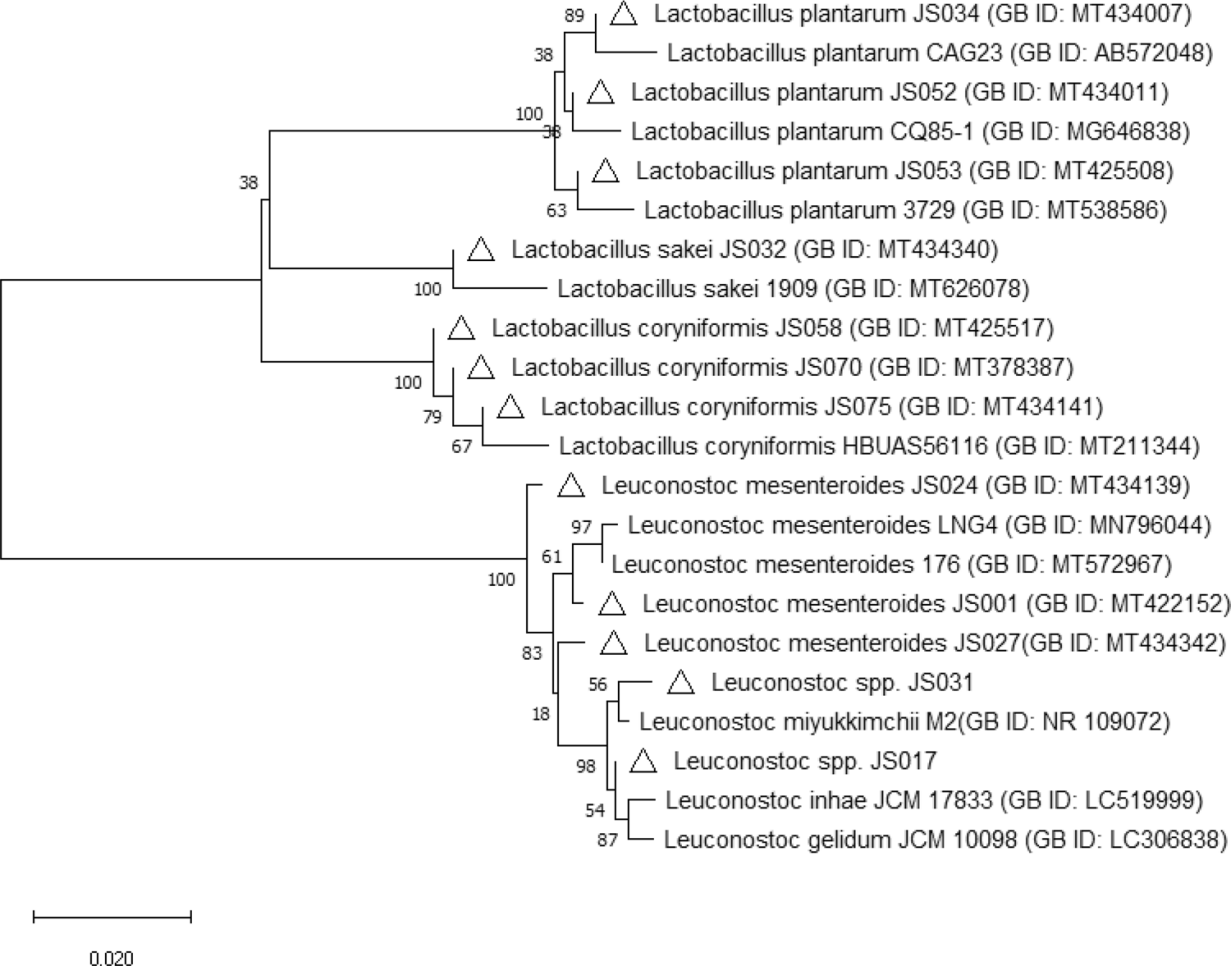
Phylogenetic tree based on 16S rRNA gene sequences showing the position of 12 LAB isolates. (Asterisk) The unrooted tree was constructed with the neighbour-joining method with bootstrap test (2000 replicates) (Felsenstein 1985; Kumar et al. 2018; Saitou and Imanishi 1989; Tamura et al. 2004). Δ—refers to the LAB strains isolated in this paper

Besides, in order to identify the isolated strains and confirm that they belonged to specific species, molecular phylogeny analysis was performed, and a phylogenetic tree was constructed based on 16S rRNA sequences by neighbour-joining (Fig. 4) (Saitou and Imanishi 1989). Following the phylogenetic analysis, strains JS032, JS034, JS052, JS053, JS058, JS070, and JS075 were placed in the cluster making up the *Lactobacillus* genus. Within this cluster, three subgroups have been distinguished for *L. plantarum, L. coryniformis* and *L. sakei* species. The strains JS001, JS017, JS024, JS027, and JS031 were positioned in the *Leuconostoc* genus cluster, which was divided into two main subgroups, including species like as *L. mesenteroides* and *Leuconostoc* spp. The conducted phylogenetic analysis also confirmed that the results of identification studies for the isolated LAB strains were correct.

## Conclusions

The strains of LAB isolated here might demonstrate great probiotic potential, both in single starter cultures or as a bacterial consortium used in the controlled fermentation of green curly kale juice. The research presented above suggests that strains of *L. mesenteroides, L. plantarum, L. sakei*, and *L. coryniformis* are valuable isolates, significantly affecting the quality, safety, and health properties of fermented green curly kale juice under controlled conditions. Overall, *L. plantarum* isolates were characterized by the best properties in terms of antimicrobial activity, antibiotic susceptibility, and survival at low pH values or different NaCl and bile salt concentrations among the tested strains. The strains of *L. plantarum* (JS034, JS052, and JS053) relatively inhibited the growth of most studied microorganisms, in particular *B. subtilis, S. aureus, M. luteus, P. aeruginosa*, and *S.* Enteritidis. Moreover, antibiotic susceptibility was satisfactory and the resistance profile was characteristic for the LAB group, which are typically resistant against vancomycin, kanamycin, and gentamicin. Furthermore, when the above-mentioned strains were grown under simulated conditions of the gastrointestinal tract, they were found likely to survive there and colonize the intestine. In conclusion, these isolates are excellent candidates for further examination in vivo, to determine their potential health properties, and also in green curly kale fermentation processes, to evaluate their technological efficiency as novel probiotic starter cultures.
