# Correction to: Probiotic potential of lactic acid bacteria obtained from fermented curly kale juice

**DOI:** 10.1007/s00203-021-02340-4

**Published:** 2021-04-28

**Authors:** Julia Szutowska, Daniela Gwiazdowska

**Affiliations:** Department of Natural Science and Quality Assurance, Institute of Quality Science, Poznań University of Economics and Business, Poznań, Poland

## Correction to: Archives of Microbiology (2021) 203:975–988 10.1007/s00203-020-02095-4

In the original article, the legend for Fig. 3 is missing. The missing information is given as follows.

Figure 3 NaCl, acid and bile salt tolerance. **a** *L. mesenteroides* JS001*, ***b** *Leuconostoc* spp. JS017, **c** *L. mesenteroides* JS024, **d** *L. mesenteroides* JS027, **e** *Leuconostoc* spp. JS031*,* **f** *L. sakei* JS032, **g** *L. plantarum* JS034, **h** *L. plantarum* JS052, **i** *L. plantarum* JS053, **j** *L. coryniformis* JS058, **k** *L. coryniformis* JS070, **l** *L. coryniformis* JS075

